# Understanding the Long-Term Effects of Inverted-T-Abdominoplasty on Quality of Life: Insights from Post-Bariatric Surgery Patients

**DOI:** 10.3390/life15020214

**Published:** 2025-01-31

**Authors:** Adrian Matthias Vater, Jasmin Fietz, Lennart Erik Schultze-Mosgau, Philipp Edmund Lamby, Klaus Erich Gerauer, Karsten Schmidt, Rafael Gregor Jakubietz, Michael Georg Jakubietz

**Affiliations:** 1 Department of Plastic, Aestethic, Hand and Reconstructive Surgery, Medizincampus Niederbayern (University of Regensburg), General Hospital Passau, Innstraße 76, 94032 Passau, Germany; 2 Department of Trauma-, Hand-, Plastic and Reconstructive Surgery, University Hospital Würzburg, Oberdürrbacher Straße 6, 7080 Würzburg, Germany; 3 Department of General-, Visceral-, Thoracal-, Vascular-, Pediatric- and Obesity Surgery, Medizincampus Niederbayern (University of Regensburg), General Hospital Passau, Innstraße 76, 94032 Passau, Germany

**Keywords:** plastic surgery, massive weight loss, body contouring, fleur-de-lis-abdominoplasty, body image, skin excess

## Abstract

Background: Body contouring procedures following massive weight loss (MWL) are in increasing demand, with abdominoplasty emerging as one of the most frequently sought options to address excess skin and contour issues. This study comprehensively examines long-term changes in Quality of Life (QoL) following abdominoplasty in individuals who have undergone MWL. Methods: In this retrospective, single-center study, 54 post-bariatric patients who had experienced MWL and subsequently underwent abdominoplasty were included. The mean age was 50.8 years. Multiple aspects of QoL were assessed using a specially designed questionnaire administered pre- and postoperatively. Inclusion criteria included a BMI < 35 and a previous bariatric procedure. Results: The physical component score (PCS-12) demonstrated significant improvement both early and late postoperatively, whereas the mental component score (MCS-12) showed no significant change. Vitality and self-acceptance exhibited significant improvements in both the short and long term. In contrast, body contact, sexuality, and self-esteem showed no long-term improvement post-surgery. Depression scores (PHQ-4) had no positive impact on long-term QoL. Body function (X-SMFA) showed significant long-term improvement, though the impairment score revealed no significant change. Conclusions: Consistent with prior research, abdominoplasty following MWL leads to positive physical outcomes. However, the impact on body image is heterogeneous, with no sustained improvement observed in some psychosocial dimensions. As depression and body impairment scores also do not show lasting improvement, patients with expectations of broad QoL enhancements should be carefully selected and assessed for potential multidisciplinary treatment.

## 1. Introduction

Morbid obesity has become an escalating public health challenge in the Western world, correlating with significant comorbidities such as cardiovascular disease, diabetes, and mobility issues, which collectively place a substantial burden on healthcare systems. In response, there has been a rising demand for bariatric interventions, including gastric bypass (Roux-en-Y) and sleeve gastrectomy, which are effective at promoting and maintaining massive weight loss (MWL). MWL is generally defined as a 50% or greater reduction in weight in excess of a patient’s ideal Body Mass Index (BMI), providing substantial health benefits for patients with morbid obesity [1,2].

However, while bariatric procedures are highly effective in terms of weight reduction, they also introduce new challenges, particularly related to excess skin. This phenomenon, which can cause significant physical discomfort and psychological distress, negatively impacts QoL. Patients often experience symptoms such as intertrigo and body image concerns, further compounded by decreased self-esteem. They also report significant interference with daily activities, including dressing, walking, and sexual activity, as patients may face discomfort, restricted mobility, and diminished confidence in intimate settings [3,4].

These issues are most commonly localized in the trunk and extremities, making these regions primary targets for body contouring surgeries [5].

Abdominoplasty, especially the inverted-T technique involving both horizontal and vertical skin removal, has thus become one of the most frequently performed body contouring procedures to address the complications of excess skin due to MWL after bariatric surgery. This technique is particularly valued for its ability to effectively address the upper and lower abdomen in both vertical and transverse orientation, providing a more comprehensive contour for MWL patients [6]. While existing literature supports short-term QoL improvements following body contouring procedures, the long-term effects, particularly beyond nine years postoperatively, remain underexplored [7,8,9,10,11,12].

Given the complexity of MWL patients’ psychological and physical adaptation over time, understanding the long-term outcomes of abdominoplasty is crucial for shaping realistic patient expectations and improving postoperative care. Long-term QoL changes encompass not only the immediate postoperative recovery but also the stabilization and adaptation processes, which include body image acceptance, sustained self-esteem, and continued satisfaction with physical function. Unfortunately, there is currently limited validated data on the effects of these procedures on QoL over extended periods.

This study seeks to address this gap by evaluating the short- and long-term QoL outcomes following inverted-T-abdominoplasty in MWL patients, with a follow-up period extending up to nine years. This extended follow-up allows for unique insights into both the initial postoperative improvements and the enduring physical and psychological outcomes. The findings of this study will add to the literature by elucidating the sustained benefits of body contouring surgeries, particularly in the areas of body image, self-esteem, and overall patient satisfaction.

## 2. Material and Methods

In this retrospective, single-center study, we contacted all patients who underwent abdominoplasty following MWL at the University Hospital Würzburg between 2009 and 2018. All surgeries were performed by a consistent team of surgeons using the inverted-T-abdominoplasty technique, characterized by incisions along both the horizontal and vertical planes of the abdomen to address skin excess comprehensively.

Data collected included patient-related information, such as age, sex, and total weight loss. Inclusion criteria included patients who achieved MWL following either sleeve gastrectomy or gastric bypass (Roux-en-Y) procedures and subsequently opted for inverted-T-abdominoplasty to manage excess skin. Exclusion criteria were implemented to maintain the study’s rigor, excluding patients with mental health diagnoses, language comprehension barriers, or notably inconsistent questionnaire responses.

To assess patients’ QoL postoperatively, we used the “Bavarian Plastic Surgery Questionnaire”, a validated tool that incorporates four healthcare scores commonly used in clinical settings to offer a multidimensional view of QoL [13,14,15,16,17,18,19]. This approach provides a robust framework for examining the various QoL dimensions in post-MWL patients and contributes valuable data to guide future clinical practices in body contouring post-bariatric surgery.

The following scores were included:

- Short Form Health Survey-12 (SF-12): Evaluates eight health dimensions including physical functioning, pain, vitality, and mental health, providing both a physical and mental component score standardized to a mean of 50 with a standard deviation of 10.
- Dresden Body Image Score-35 (DKB-35): Measures five aspects of body image—vitality, self-acceptance, body contact, sexuality, and self-esteem—allowing patients to rate their agreement on a scale from 1 (strongly agree) to 5 (strongly disagree).
- Patient Health Questionnaire (PHQ-4): Assesses symptoms of depression, offering scores from 0 to 3 per item, up to a maximum of 12, to evaluate the frequency and severity of depressive symptoms.
- Short Musculoskeletal Function Assessment (X-SMFA): Assesses musculoskeletal function, focusing on functional limitations and impairment, which are common concerns for patients with significant weight changes.

Data collection covered both preoperative and postoperative QoL dimensions, including functional, mental health, sexuality, and body image aspects. Questionnaires were administered to the patients in 2022. Fifty-four participants completed the questionnaires and were categorized into two groups based on the timing of surgery: Group 1 (“early”, 2014–2018) with short-term effects (4–8 years postoperative) and Group 2 (“late”, 2009–2013) with long-term effects (9–13 years postoperative).

Ethical approval was obtained from the local ethics committee (ref. 2024091001), and all participants provided written consent. Statistical analysis was conducted using IBM SPSS Statistics Version 29.0.2.0 (20), with t-tests applied for continuous variables and a 95% confidence interval. Statistical significance was set at *p* < 0.05.

## 3. Results

Of the 54 participants included, 45 were female and 9 were male, with an overall mean age of 50.81 years. Group 1 (“early”) included 30 participants with a mean age of 50.8 years, while Group 2 (“late”) comprised 24 participants with a mean age of 50.9 years (Table 1). Both groups were comparable in BMI due to inclusion criteria. The median weight loss across all patients was 57.0 kilograms (kg), with female patients losing an average of 53.8 kg and male patients collective achieving a weight loss of 73.2 kg.

**Figure 1:**
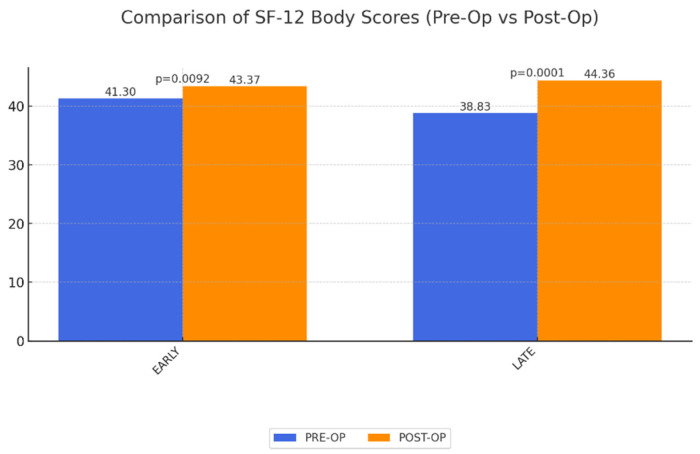
Significant improvement in physical QoL (SF-12 body) early and late postoperatively after abdominoplasty as a result of MWL. A value of 50 corresponds to population average.

**Figure 2:**
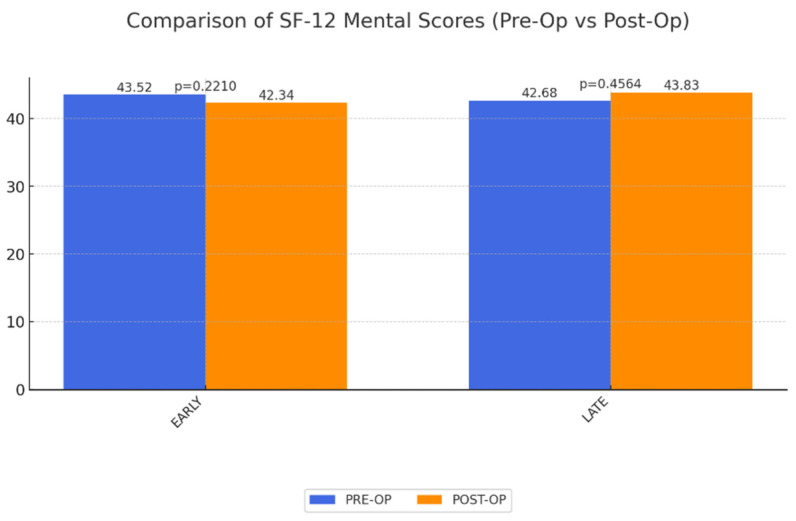
Mean results for mental QoL (SF-12 mental) early and late postoperatively after abdominoplasty as a result of MWL. A value of 50 corresponds to population average. No significance.

SF-12 Physical and Mental Component Scores: Analysis showed a statistically significant improvement in physical QoL in both groups when compared to preoperative levels. These physical improvements likely facilitate better mobility and reduced discomfort, which are critical for the daily lives of MWL patients. Conversely, mental QoL showed no significant short-term and long-term change (Figure 1 and Figure 2).

**Figure 3:**
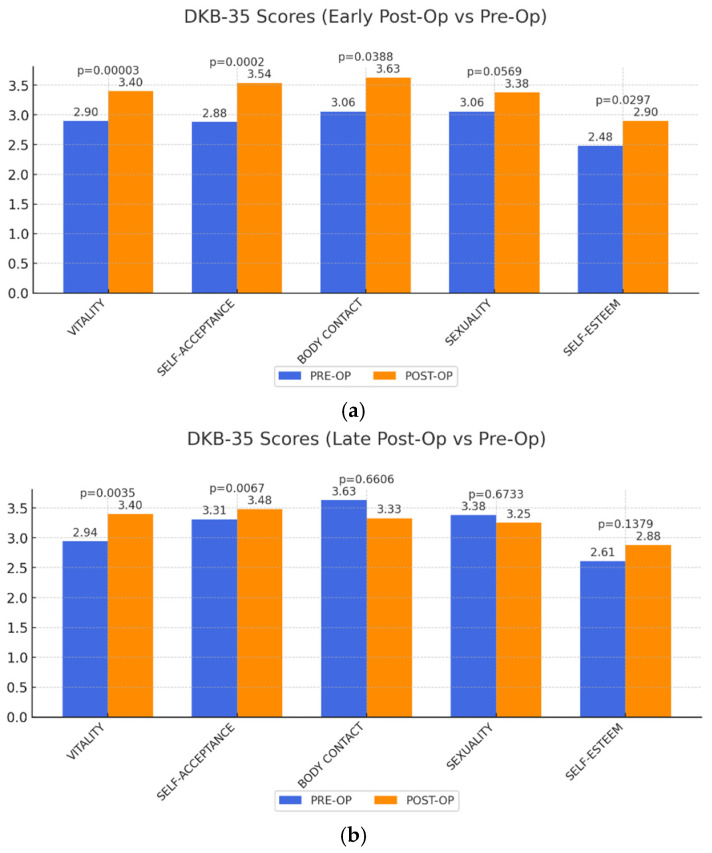
(**a**,**b**) Change in five dimensions of DKB-35 questionnaire early and late postoperatively after abdominoplasty as a result of MWL. Maximum achievable value is 5.

DKB-35 Body Image Scores: In body image aspects, we observed significant improvements in vitality and self-acceptance in both early and late postoperative stages. Body contact and self-esteem showed initial gains but diminished in the long term. Sexuality, however, remained largely unchanged (Figure 3).

Vitality and self-acceptance show significant improvement short-and long-term. Body contact and self-esteem show significant short-term but no long-term improvement. Sexuality does not show significant improvement at all.

**Figure 4:**
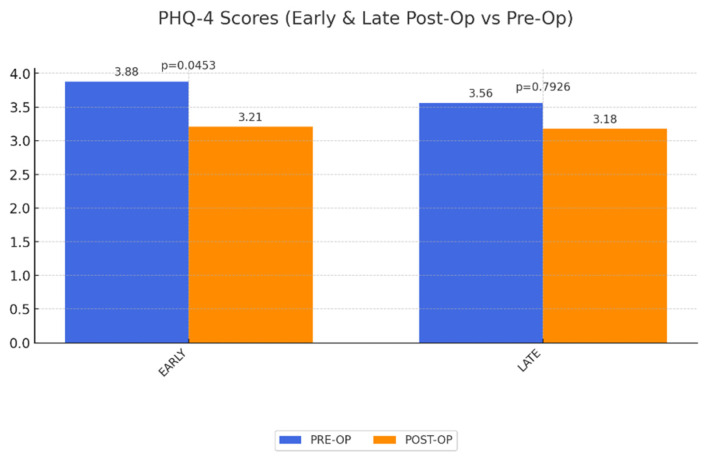
Change of QoL concerning depression and anxiety disorder score (PHQ-4) early and late postoperatively after abdominoplasty as a result of MWL. Maximum achievable value is 4. Significant improvement short-term, no significance long-term.

PHQ-4 Depression Scores: The depression scores decreased significantly in the short term but did not sustain this improvement over time, aligning with the broader trend of diminishing long-term QoL (Figure 4).

**Figure 5:**
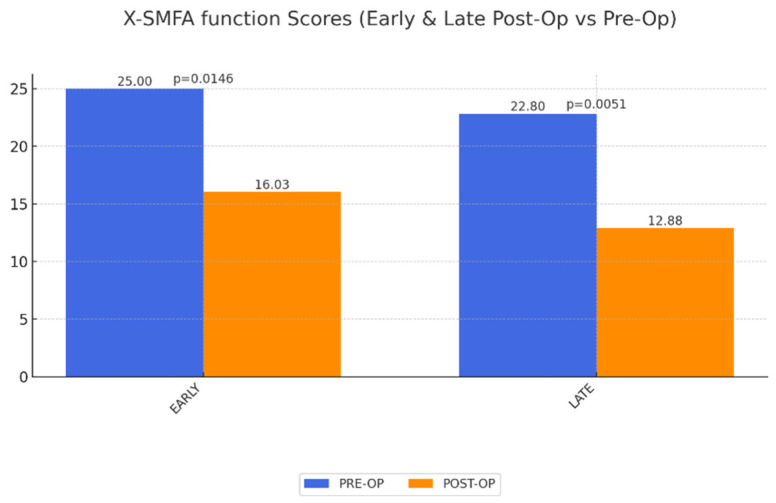
Change of QoL concerning body function (X-SMFA function) early and late postoperatively after abdominoplasty as a result of MWL. A value of 100 corresponds to worse possible function. Significant improvement of body function early and late postoperatively.

**Figure 6:**
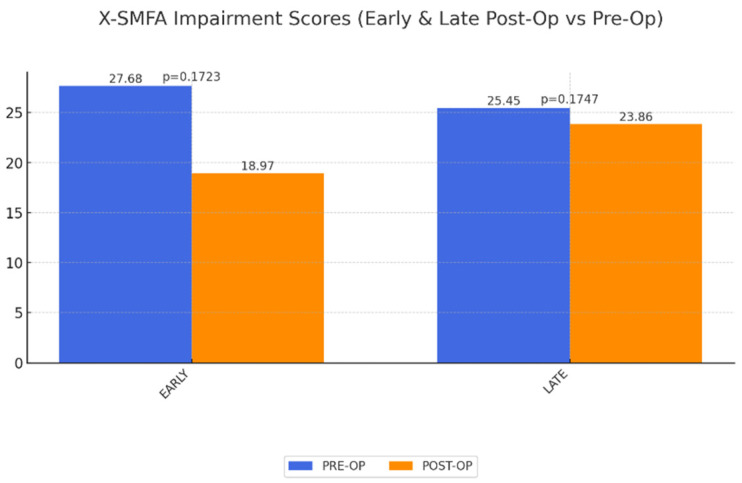
Change of QoL concerning impairment (X-SMFA impairment) early and late postoperatively after abdominoplasty as a result of MWL. A value of 100 corresponds to highest possible impairment. No significance.

X-SMFA Body Function Scores: A significant improvement in body function was observed in the long term, though the impairment scores remained unchanged, suggesting patients gained functional ability but still faced some physical limitations, potentially related to prior obesity-related health conditions (Figure 5 and Figure 6).

## 4. Discussion

The assessment of QoL from the patient’s perspective has gained increasing importance across medical disciplines, including plastic surgery. This shift reflects the growing recognition of patient-reported outcomes as key measures of treatment success and overall well-being [20].

Historically, surgical success was evaluated using clinical metrics, such as the absence of complications or procedural effectiveness. However, the field now acknowledges that true success must include patients’ perceptions of their QoL after treatment. This shift is particularly relevant in bariatric and post-bariatric surgery, where the challenges of morbid obesity demand comprehensive and multidimensional solutions.

Bariatric surgery aims not only to achieve substantial weight loss but also to improve related comorbidities and enhance overall QoL. While these procedures often lead to significant improvements in physical health, psychological well-being, and social interactions, our understanding of their long-term impact on QoL following post-bariatric plastic surgery remains limited. Bridging this knowledge gap is essential, as many patients pursue such surgeries hoping for lasting benefits.

Our study provides insights into the multifaceted QoL outcomes in patients undergoing abdominoplasty after MWL.

Consistent with previous studies, significant improvements, particularly in physical QoL, were observed in various domains [21]. However, some effects diminished over time, highlighting the need for a nuanced understanding of how QoL evolves in this population. While surgery can address physical concerns, its impact is shaped by complex interactions between physical and psychological factors.

Interestingly, long-term physical improvements such as enhanced vitality and self-acceptance persisted even in the absence of significant changes in depression or mental health. Patients reported increased energy levels, greater ease in physical activities, and improved body image, which collectively contributed to a more positive self-perception and lifestyle. However, challenges related to depression and mental health remained prevalent. These findings align with previous research suggesting that surgical interventions alone may not resolve pre-existing psychological issues or body image dissatisfaction. Emotional complexities and societal pressures often persist, emphasizing the need for comprehensive assessments of psychological well-being alongside physical outcomes.

Sexuality and body contact often show significant short-term improvements after MWL and abdominoplasty. Many patients report enhanced body image and increased sexual satisfaction following surgery, likely due to physical transformations, improved self-confidence, and an enhanced perception of their bodies. However, studies suggest that these improvements can be short-lived [9]. In the long term, some patients report declines in sexual satisfaction and body contact compared to preoperative levels.

This paradox can be explained by the interplay of physical and psychological factors. While MWL and body contouring can improve body image, they do not always resolve underlying psychological issues such as body dysmorphia or unresolved emotional complexities related to weight and physical appearance. Additionally, the adjustment to a new body image can be psychologically taxing, leading to renewed dissatisfaction in the long term. Paradoxically, some studies report an increase in immediate postoperative depression scores, which may stem from the emotional recalibration needed to accept the changes in body image and identity.

Factors like weight regain, psychological adaptation, and ongoing societal pressures can contribute to the decline in sexual well-being and body contact in the long term, underscoring the importance of continuous psychological support and realistic expectation management post-surgery.

The postoperative journey is marked by distinct phases. During the initial “honeymoon phase”, dramatic physical transformations often lead to euphoria, enhanced self-confidence, and optimism. However, as patients transition to the long-term postoperative phase, factors such as weight regain, postoperative complications, and persistent body image concerns may temper these initial gains [22,23].

Minor complications such as hematomas, seromas, and delayed wound healing also contribute to QoL challenges during the early postoperative weeks [24,25,26]. Managing these setbacks requires significant mental resilience. Studies report that 20–24% of patients experience weight regain of over 15% within five years post-surgery [27,28,29]. This rebound effect can lead to dissatisfaction, reinforcing the need for sustained support to maintain both physical and psychological benefits.

Additionally, the diminished capacity of skin to retract after MWL often leads to secondary sagging, despite surgical interventions. While procedures like abdominoplasty improve aesthetics, they cannot fully eliminate excess skin, creating a potential mismatch between patients’ expectations and outcomes. This disconnect can overshadow initial satisfaction and complicate adaptation to a new body image.

Paradoxically, some studies have observed an increase in immediate postoperative depression scores following aesthetic surgeries, including abdominoplasty [30]. This may stem from the emotional adjustments required to reconcile physical changes with unresolved psychological issues. Transitioning to a new self-image is a complex process that involves mental and emotional recalibration, underscoring the need for psychological support throughout recovery.

Our findings highlight the importance of addressing both psychological and physiological factors to optimize long-term QoL outcomes. While surgery can address visible manifestations of excess skin, it cannot fully resolve underlying mental health concerns or mitigate challenges like weight rebound [31,32,33,34]. Preoperative counseling and realistic expectation management are crucial to preparing patients for their postoperative journey. Comprehensive psychological evaluations could help identify those at higher risk of dissatisfaction or depression, enabling tailored interventions to support their needs.

A multidisciplinary approach involving medical professionals, psychologists, and plastic surgeons is essential for optimizing outcomes in post-bariatric patients [35]. Patient selection should consider not only anatomical criteria but also psychological readiness and expectation management. This collaborative approach fosters a holistic understanding of patient needs and enhances both physical and psychological outcomes.

Despite its valuable insights, our study has limitations. Its retrospective design introduces potential biases, particularly in self-reported QoL measures, which may be influenced by current emotions or social desirability. Additionally, the follow-up period may not fully capture the dynamic nature of QoL over time. Cultural and healthcare system differences further limit the generalizability of findings from this German cohort to other populations.

Recall bias is another inherent challenge in retrospective studies in general and also in this study, particularly in the context of bariatric surgery, where patients’ recollections of pre-surgery QoL may be influenced by their current health status and post-surgery improvements. Despite these challenges, even previous studies have successfully utilized retrospective questionnaire data to evaluate long-term QoL outcomes following bariatric surgery. For example, studies such as Karlsson et al. (2007) have effectively assessed QoL years after bariatric surgery, even without preoperative baseline data, and their findings have contributed valuable insights into the lasting impact of these interventions [36].

Future research should address these limitations through prospective, multicenter studies with longer follow-up periods and comprehensive assessments of patient-reported outcomes. Investigating the role of psychological interventions, such as counseling or support groups, could provide further insights into sustaining long-term QoL improvements for post-bariatric patients.

## 5. Conclusions

While abdominoplasty holds promise as a means to enhance QoL in patients with MWL, our study underscores the complex and evolving nature of these outcomes in this population. The interplay between physical transformations, psychological adjustments, and societal expectations is intricate and multifaceted. Moving forward, a nuanced understanding of these factors is essential to optimize treatment strategies and improve long-term patient outcomes. Emphasizing patient education, psychological support, and a multidisciplinary approach can contribute significantly to enhancing the overall QoL for individuals undergoing post-bariatric plastic surgery.

## Figures and Tables

**Table 1:** (**a**,**b**): Patient Characteristics and Scores (SF-12: Short Form Health Survey-12, DKB-35: Dresden Body Image Score-35, PHQ-4: Patient Health Questionnaire, X-SMFA: Short Musculoskeletal Function Assessment).

(**a**)					
**Group**		**EARLY**		**LATE**	
n		30		24	
male		5		4	
female		25		20	
mean age (years)		50.8		50.9	
(**b**)					
**Group**	**PRE-OP**	**EARLY**	***p***	**LATE**	***p***
SF-12 body	41.30	43.37	0.0092	38.83	0.0001
SF-12 mental	43.52	42.34	0.2210	42.68	0.4564
DKB-35 vitality	2.90	3.40	0.00003	2.94	0.0035
DKB-35 self-acceptance	2.88	3.54	0.0002	3.12	0.0067
DKB-35 body contact	3.06	3.63	0.0388	3.33	0.6606
DKB-35 sexuality	3.06	3.38	0.0569	3.20	0.6733
DKB-35 self-esteem	2.48	2.90	0.0297	2.61	0.1379
PHQ-4	3.88	3.21	0.0453	3.56	0.7926
X-SMFA function	25.00	16.03	0.0146	22.80	0.0051
X-SMFA impairment	27.68	18.97	0.1723	25.45	0.1747

## Data Availability

All patient data used were anonymized and received by the patient chart in accordance with the local ethical committee.

## References

[1] WHO (2021). Fact Sheet Obesity and Overweight.

[2] Blüher M. (2019). Obesity: Global epidemiology and pathogenesis. Nat. Rev. Endocrinol..

[3] Friedman T., O’Brien Coon D., Michaels V.J., Purnell C., Hur S., Harris D.N., Rubin J.P. (2010). Fleur-de-Lis abdominoplasty: A safe alternative to traditional abdominoplasty for the massive weight loss patient. Plast Reconstr. Surg..

[4] Losco L., Roxo A.C., Roxo C.W., de Sire A., Bolletta A., Cuomo R., Grimaldi L., Cigna E., Roxo C.D.P. (2022). Helix Thigh Lift. A Novel Approach to Severe Deformities in Massive Weight Loss Patients. J. Investig. Surg..

[5] Shermak M.A., Chang D., Magnuson T.H., Schweitzer M.A. (2006). An outcomes analysis of patients undergoing body contouring surgery after massive weight loss. Plast Reconstr Surg..

[6] Mitchell R.T., Rubin J.P. (2014). The Fleur-De-Lis abdominoplasty. Clin. Plast Surg..

[7] Kitzinger H.B., Abayev S., Pittermann A., Karle B., Kubiena H., Bohdjalian A., Langer F.B., Prager G., Frey M. (2012). The prevalence of body contouring surgery after gastric bypass surgery. Obes Surg..

[8] Van der Beek E.S., Geenen R., de Heer F.A., van der Molen A.B.M., van Ramshorst B. (2012). Quality of life long-term after body contouring surgery following bariatric surgery: Sustained improvement after 7 years. Plast. Reconstr. Surg..

[9] Toma T., Harling L., Athanasiou T., Darzi A., Ashrafian H. (2018). Does Body Contouring After Bariatric Weight Loss Enhance Quality of Life? A Systematic Review of QOL Studies. Obes Surg..

[10] van der Beek E.S., Te Riele W., Specken T.F., Boerma D., van Ramshorst B. (2010). The impact of reconstructive procedures following bariatric surgery on patient well-being and quality of life. Obes. Surg..

[11] Lazar C.C., Clerc I., Deneuve S., Auquit-Auckbur I., Milliez P.Y. (2009). Abdominoplasty after major weight loss: Improvement of quality of life and psychological status. Obes. Surg..

[12] Stuerz K., Piza H., Niewrmank H., Kinz J.F. (2008). Psychosocial impact of abdominoplasty. Obes. Surg..

[13] Buchwald H., Avidor Y., Braunwald E., Jensen M.D., Pories W., Fahrbach K., Schoelles K. (2004). Bariatric surgery: A systematic review and meta-analysis. JAMA.

[14] Courcoulas A.P., Yanovski S.Z., Bonds D., Eggerman T.L., Horlick M., Staten M.A., Arterburn D.E. (2014). Long-term outcomes of bariatric surgery: A National Institutes of Health symposium. JAMA Surg..

[15] Schmidt K., Jakubietz M.G., Gilbert F., Hausknecht F., Meffert R.H., Jakubietz R.G. (2019). Quality of Life after Flap Reconstruction of the Distal Lower Extremity: Is There a Difference Between a Pedicled Suralis Flap and a Free Anterior Lateral Thigh Flap?. Plast. Reconstr. Surg. Glob. Open.

[16] Drixler K., Morfeld M., Glaesmer H., Brähler E., Wirtz M.A. (2020). Validierung der Messung gesundheitsbezogener Lebensqualität mittels des Short-Form-Health-Survey-12 (SF-12 Version 2.0) in einer deutschen Normstichprobe. Z. Psychosom. Med. Psychother..

[17] Kazlauskas E., Gelezelyte O., Kvedaraite M., Ajdukovic D., Johannesson K.B., Böttche M., Bondjers K., Dragan M., Figueiredo-Braga M., Grajewski P. (2023). Psychometric properties of the Patient Health Questionnaire-4 (PHQ-4) in 9230 adults across seven European countries: Findings from the ESTSS ADJUST study. J. Affect Disord..

[18] Pöhlmann K., Roth M., Brähler E., Joraschky P. (2014). Der Dresdner Körperbildfragebogen (DKB-35): Validierung auf der Basis einer klinischen Stichprobe. PPmP-Psychother. Psychosom. Med. Psychol..

[19] Wollmerstedt N., Faller H., Ackermann H., Schneider J., Glatzel M., Kirschner S., König A. (2006). Evaluierung des XSMFA-D an Patienten mit Erkrankungen des Bewegungsapparates und operativer oder konservativer stationärer Therapie [Evaluation of the Extra Short Musculoskeletal Function Assessment questionnaire XSMFA-D in patients with musculoskeletal disorders and surgical or medical in-patient treatment]. Rehabilitation.

[20] Haraldstad K., Wahl A., Andenæs R., Andersen J.R., Andersen M.H., Beisland E., Borge C.R., Engebretsen E., Eisemann M., Halvorsrud L. (2019). LIVSFORSK network. A systematic review of quality of life research in medicine and health sciences. Qual. Life Res..

[21] Aitzetmüller M.M., Raschke L., Klietz M.L., Kueckelhaus M., Hirsch T., Wiebringhaus P., Harati K. (2022). After weight loss, what skin removal procedure has the most effect using Body Q metrics?. Surg. Obes. Relat. Dis..

[22] Gilmartin J., Long A.F., Soldin M. (2015). Identity transformation and a changed lifestyle following dramatic weight loss and body contouring surgery. J. Health Psychol..

[23] Lynch A. (2016). When the honeymoon is over, the real work begins: Gastric bypass patients’ weight loss trajectories and dietary change experiences. Soc. Sci. Med. 1982.

[24] De Paep K., Van Campenhout I., Van Cauwenberge S., Dillemans B. (2021). Post-bariatric Abdominoplasty: Identification of Risk Factors for Complications. Obes. Surg..

[25] Chaouat M., Levan P., Lalanne B., Buisson T., Nicolau P., Mimoun M. (2000). Abdominal dermolipectomies: Early postoperative complications and long-term unfavorable results. Plast. Reconstr. Surg..

[26] Brito Í.M., Meireles R., Baltazar J., Brandão C., Sanches F., Freire-Santos M.J. (2020). Abdominoplasty and Patient Safety: The Impact of Body Mass Index and Bariatric Surgery on Complications Profile. Aesth. Plast Surg..

[27] Tolvanen L., Christenson A., Surkan P.J., Lagerros Y.T. (2022). Patients’ Experiences of Weight Regain After Bariatric Surgery. Obes. Surg..

[28] Brissman M., Beamish A.J., Olbers T., Marcus C. (2021). Prevalence of insufficient weight loss 5 years after Roux-en-Y gastric bypass: Metabolic consequences and prediction estimates: A prospective registry study. BMJ Open.

[29] Voorwinde V., Steenhuis I.H., Janssen I.M., Monpellier V.M., van Stralen M.M. (2020). Definitions of Long-Term Weight Regain and Their Associations with Clinical Outcomes. Obes. Surg..

[30] Jones H.E., Faulkner H.R., Losken A. (2022). The Psychological Impact of Aesthetic Surgery: A Mini-Review. Aesthet. Surg. J. Open Forum.

[31] Peterhänsel C., Petroff D., Klinitzke G., Kersting A., Wagner B. (2013). Risk of completed suicide after bariatric surgery: A systematic review. Obes. Rev..

[32] Bhatti J.A., Nathens A.B., Thiruchelvam D., Grantcharov T., Goldstein B.I., Redelmeier D.A. (2016). Self-harm emergencies after bariatric surgery: A population-based cohort study. JAMA Surg..

[33] Morgan D.J., Ho K.M. (2017). Incidence and risk factors for deliberate self-harm, mental illness, and suicide following bariatric surgery. Ann. Surg..

[34] de Zwaan M., Georgiadou E., Stroh C.E., Teufel M., Köhler H., Tengler M., Müller A. (2014). Body image and quality of life in patients with and without body contouring surgery following bariatric surgery: A comparison of pre- and post-surgery groups. Front Psychol..

[35] Ivezaj V., Grilo C.M. (2018). The complexity of body image following bariatric surgery: A systematic review of the literature. Obes. Rev..

[36] Karlsson J., Taft C., Rydén A., Sjöström L., Sullivan M. (2007). Ten-year trends in health-related quality of life after surgical and conventional treatment for severe obesity: The SOS intervention study. Int. J. Obes..

